# Rationale, Modelle und Wirkung arbeitsplatznaher psychotherapeutischer Angebote

**DOI:** 10.1007/s00103-024-03892-8

**Published:** 2024-05-28

**Authors:** Peter Angerer, Harald Gündel, Christoph Kröger, Eva Rothermund

**Affiliations:** 1 Institut für Arbeits‑, Sozial- und Umweltmedizin, Centre for Health and Society, Medizinische Fakultät und Universitätsklinikum Düsseldorf, Heinrich-Heine-Universität Düsseldorf, Moorenstraße 5, 40225 Düsseldorf, Deutschland; 2 Klinik für Psychosomatische Medizin und Psychotherapie, Universitätsklinikum Ulm, Ulm, Deutschland; 3 Abteilung Klinische Psychologie und Psychotherapie, Institut für Psychologie, Universität Hildesheim, Hildesheim, Deutschland

**Keywords:** Psychotherapeutische Sprechstunde am Arbeitsplatz, Psychische Erkrankungen, Arbeitsstress, Prävention, Behandlung, Psychotherapeutic Consultation at the Workplace, Mental illness, Work stress, Prevention, Treatment

## Abstract

Psychische Erkrankungen und Verhaltensstörungen sind auch in der erwerbstätigen Bevölkerung sehr häufig. Sie betreffen bis zu ein Drittel der Beschäftigten pro Jahr und gehen mit erheblichem Leidensdruck, dem Risiko der Chronifizierung und des Ausscheidens aus der Erwerbstätigkeit einher. Wirtschaftlich verursachen sie hohe Kosten. Um diese Folgen abzumildern und die Genesungschancen zu erhöhen, sind eine rasche Diagnostik, ggf. eine frühzeitige adäquate Therapie und – über die übliche Vorgehensweise der Psychotherapie hinaus – eine Beachtung der arbeitsbedingten Ursachen von entscheidender Bedeutung.

Die Psychotherapeutische Sprechstunde am Arbeitsplatz (PT-A) versucht, diesen Anforderungen gerecht zu werden. Sie bietet kurzfristig und in Arbeitsplatznähe psychotherapeutische Hilfe für psychisch belastete Beschäftigte an, leistet – je nach Problemlage – Beratung, Diagnostik, Prävention und kurzzeitige bzw. überbrückende Therapie und unterstützt bei der Wiedereingliederung nach längerer psychischer Erkrankung. Hilfreich ist eine enge Kooperation mit dem betriebsärztlichen Dienst, der die PT‑A zu Rate zieht, an sie überweist, Kenntnisse zur betrieblichen Situation beisteuern und ggf. die Wiedereingliederung begleiten kann. Die Finanzierung übernimmt häufig der Betrieb, ggf. aber auch Krankenkassen in Modellen der integrierten Versorgung.

In dem vorliegenden Beitrag werden zunächst die Geschichte und die Grundlagen von PT‑A sowie die Rolle von Arbeitsstress bei der Entstehung psychischer und psychosomatischer Störungen beschrieben. Die Umsetzung der PT‑A wird an 2 Beispielen skizziert. Abschließend wird die aktuelle Studie „Frühe Intervention am Arbeitsplatz“ (friaa) kurz vorgestellt, auf die sich mehrere Artikel in diesem Themenheft beziehen.

## Abstract (en)

Mental illnesses and behavioral disorders are very common among the working population, affecting up to a third of employees each year, and are associated with great suffering, the risk of chronicity, and the loss of employment. Economically, mental illnesses cause high costs. In order to mitigate these consequences and increase the chances of recovery, rapid diagnosis, early and appropriate treatment where necessary, and—over and above the usual psychotherapy approach—attention to the work-related causes are of crucial importance.

Psychotherapeutic Consultation at the Workplace (PT-A) attempts to meet these requirements. It offers psychotherapeutic help at short notice and close to the workplace for employees suffering from mental stress; provides (depending on the problem) counseling, diagnostics, prevention, and short-term or bridging therapy; and supports reintegration after a longer period of mental illness. It is helpful to cooperate closely with the company medical service, which consults the PT‑A, refers employees to it, provides information on the company situation, and can support reintegration if necessary. Funding is often provided by the company but can also be provided by health insurance companies in integrated care models.

This article begins by describing the history and principles of PT‑A and the role of work stress in the development of psychological and psychosomatic disorders. The implementation of PT‑A is then outlined using two examples. Finally, the current study “Early Intervention at the Workplace” (“Frühe Intervention am Arbeitsplatz” [friaa]), to which several articles in this special issue refer, is briefly presented.

## Hintergrund

Nach den Daten des Bundesgesundheitssurveys 1998 und der Studie zur Gesundheit Erwachsener in Deutschland 2011 (DEGS1) sind 33,3 % der Frauen und 22,0 % der Männer in Deutschland im Laufe eines Jahres von einer klinisch relevanten psychischen Störung betroffen [1]. Während laut diesen bevölkerungsbezogenen Studien die Häufigkeit psychischer Störungen weitgehend unverändert geblieben ist [1], zeigen die Statistiken der gesetzlichen Krankenkassen und der Deutschen Rentenversicherung eine stetige Zunahme der entsprechenden Arbeitsunfähigkeits(AU)-Zeiten [2]. Mögliche Gründe sind der Einsatz besserer Diagnostik, mehr Versorgungsangebote sowie die partielle Enttabuisierung psychischer Störungen (siehe dazu auch Heft 4/2023 – Public Mental Health), aber auch Belastungen durch aktuelle Krisen und die Veränderungen am Arbeitsmarkt mit höheren Anforderungen an die Leistungsfähigkeit der Beschäftigten [3].

Psychische Störungen gehen im Schnitt mit einer deutlich höheren Anzahl an AU-Tagen pro Erkrankungsfall einher als viele somatische Erkrankungen [2]. Neben volkswirtschaftlichen und betrieblichen Auswirkungen wie Produktionsausfallkosten in 2‑stelliger Milliardenhöhe [4] hat dies v. a. für die betroffene Person gravierende Folgen. So kommt zu dem Leiden durch die Erkrankung an sich u. U. der Wegfall wichtiger Funktionen von Arbeit hinzu, wie etwa soziale Anerkennung, die Anwendung von Kompetenzen und die Strukturierung des Alltags, welche die allgemeine Lebenszufriedenheit maßgeblich beeinflussen (z. B. [5]). Je länger eine Arbeitsunfähigkeit andauert, desto höher ist das Risiko einer Chronifizierung psychischer Störungen (s. unten) und einer Frühberentung [6]. Des Weiteren erhöhen psychische Störungen das Risiko, arbeitslos zu werden, und vermindern die Chance, aus der Arbeitslosigkeit heraus wieder eine Arbeitstätigkeit zu finden [6]. Arbeit kann aber nicht nur gesundheitsförderliche Funktionen haben, sondern durch Arbeitsstress auch psychisch krank machen (s. unten). Insofern besteht im Hinblick auf die Gestaltung gesunder und gesundheitsförderlicher Arbeitsbedingungen ebenfalls besonderer Handlungsbedarf. Als Ansatzpunkt, um diese Problematiken zu bearbeiten, bietet sich die Schnittstelle zwischen dem Gesundheitssystem in Deutschland mit präventiven, kurativen und rehabilitativen Angeboten und der Arbeitswelt an, in der diese Angebote gebraucht werden.

Das hier vorliegende Heft des Bundesgesundheitsblatts hat seinen Schwerpunkt in individualpräventiven und -rehabilitativen Maßnahmen an dieser Schnittstelle. Die psychotherapeutische Sprechstunde am Arbeitsplatz (PT-A), also in Arbeitsnähe, ist ein innovatives und potenziell sehr wirkungsvolles Instrument, das sich über Jahre in verschiedensten Modellen und verstreut über einzelne Unternehmen in Deutschland entwickelt hat, dessen Potenzial aber bei Weitem nicht ausgeschöpft wird. Historie, Rationale, möglicher Nutzen, Charakteristika dieses Angebots, Besonderheit seiner Positionierung im Setting Arbeitsplatz und aktuelle Forschungsbemühungen werden in der folgenden Übersicht dargestellt.

## Geschichte und Grundlagen psychotherapeutischer Sprechstunden am Arbeitsplatz

Die psychosomatische bzw. psychotherapeutische Sprechstunde im Betrieb, hier einheitlich mit „psychotherapeutische Sprechstunde am Arbeitsplatz (PT-A)“ bezeichnet, ist u. W. in Deutschland erstmals vor ca. 15–20 Jahren entstanden. Es gab einzelne psychosomatische (Stuttgart, später Ulm) bzw. psychiatrische Kliniken (Hamburg) in Deutschland, die ein solches Versorgungsangebot erstmals entwickelt haben. Dem zugrunde liegen Erfahrungen des in den „Psych*-Fächern“^1^ häufig praktizierten Konsiliar‑/Liaisonmodells: Die Grundidee ist hier, nicht zu warten, bis geeignete Patient:innen von sich aus den Weg zu fachlich geeigneten Expert:innen suchen und finden. Stattdessen gehen die Expert:innen an die Orte, wo sie direkt für die jeweiligen Patient:innen erreichbar sind. Gerade vor dem Hintergrund der vor 20 Jahren noch stärkeren Stigmatisierung psychischer und psychosomatischer Erkrankungen war von Beginn an – ähnlich wie bei der interdisziplinären Schmerztherapie – die enge Zusammenarbeit der Psych*-Expert:innen mit den jeweils zuständigen Betriebsmediziner:innen ein essentieller Bestandteil der Sprechstunde im Betrieb. Die einzelnen Psych*-Expert:innen konnten regelmäßig Gesprächstermine z. B. in Räumen des betriebsmedizinischen Dienstes anbieten, von außen war nicht erkennbar, wer auf dem Betriebsgelände zum Betriebsarzt zur Vorsorge oder zur psychosomatischen Erstberatung ging. Der:die Betriebsmediziner:in konnte im Rahmen dieser Zusammenarbeit konkret und unkompliziert Patient:innen an die psychosomatische Beratung vermitteln.

Das zentrale Ziel war und ist, ein möglichst niedrigschwelliges Versorgungsangebot machen zu können – im Fall der psychosomatischen Sprechstunde im Betrieb spezifisch für Menschen, die einerseits einen Bedarf an Erstberatung aufweisen und eine entsprechende Motivation besitzen, andererseits aber Hemmnisse erleben, eine solche Erstberatung realisieren zu können (innere Hemmnisse: z. B. Schamgefühle; äußere Hemmnisse: z. B. schwerer Zugang zu niedergelassenen Psychotherapeut:innen). Diese Konstellation führt zu den klinisch oft erlebten, sehr späten Zugängen in eine indizierte (Kurzzeit‑)Psychotherapie. Dies ist nicht nur für die einzelnen Betroffenen im Hinblick auf persönliches Leid und verminderte Lebensqualität, sondern auch für die jeweiligen Arbeitgeber im Hinblick auf entstehende AU-Zeiten sehr bedauerlich. Dabei wurde häufig genug gezeigt, dass schon nach 8–13 Sitzungen Einzelpsychotherapie („good enough dose“) ca. 50 % der Patient:innen profitieren [7].

Die der PT‑A zugrunde liegende Idee lässt sich wie folgt veranschaulichen: Prävention und frühe Behandlung sind in der Regel wirksamer als die Behandlung einer bereits voll ausgebildeten Erkrankung: Beispielsweise ist es bei einer Sehnenreizung durch Überbeanspruchung völlig nachvollziehbar, dass weiterer dauerhafter Gebrauch ohne entsprechende Behandlung zu einer schrittweisen Chronifizierung der Sehnenentzündung führen kann. Am Anfang steht dabei eine lediglich funktionelle, gut reversible Entzündungsreaktion, im Laufe der weiteren Beanspruchung ohne adäquate Therapie kommt es schrittweise zu einem bindegewebigen Umbau, der dann nicht mehr ohne Weiteres rückgängig gemacht werden kann. Alexander Mitscherlich, einer der Gründungsväter der deutschen psychosomatischen Medizin, sprach mit Blick auf viele stressassoziierte Erkrankungen von einer Zerreißung des „psycho-somatischen Simultan-Geschehens“ [8]: Schrittweise wird eine zunächst reine Störung der Funktion auch zu einer strukturellen, nicht mehr veränderbaren Pathologie umgebaut (gilt z. B. auch für Hypertonie und Arteriosklerose).

Psychische Prozesse, d. h. die vielfältigen Funktionen des zentralen Nervensystems, sind gegenüber einer Sehnenentzündung sicher komplexer und viel schwieriger zu fassen – andererseits gelten auch hier ähnliche Mechanismen: Ohne frühzeitige adäquate Therapie besteht die Gefahr einer Chronifizierung und langsamen Verschlechterung der zugrunde liegenden psychischen bzw. psychosomatischen Erkrankung, vermutlich unter Einschluss der neurobiologisch mit den Beschwerden korrelierenden Grundlagen. Demgegenüber kann die frühe Behandlung zu einer schnellen Heilung oder zumindest einer signifikanten Besserung führen.

## Arbeitsstress und psychische und psychosomatische Erkrankung

Auch aus betrieblicher bzw. betriebsärztlicher Perspektive ist unbestritten und wichtig, dass gut gestaltete Arbeit viele gesundheitsförderliche Aspekte hat [9] und dass Arbeitslosigkeit krank machen kann [6]. Zusätzlich können psychosoziale Einflüsse am Arbeitsplatz das Risiko für Erkrankungen auf psychischer und körperlicher Ebene massiv erhöhen. In der Regel spielen nicht nur einzelne Faktoren wie lange Wochenarbeitszeit, hoher Zeitdruck, große Arbeitsmenge und -intensität oder fehlender Handlungsspielraum eine Rolle, sondern Kombinationen solcher Einflüsse. Das Zusammenspiel mehrerer Faktoren ist theoretisch gut begründet und in Arbeitsstressmodellen gefasst, damit mess- und beforschbar. Die wichtigsten Arbeitsstressmodelle seien hier kurz genannt:

- Hohe Anforderung, z. B. durch schwierige Aufgaben, große Arbeitsmenge oder ständigen Zeitdruck, in Kombination mit geringer Kontrolle über die Arbeit, im Sinne von Mangel an Handlungsspielraum oder Möglichkeit, zu lernen, sind die Wirkfaktoren im Anforderungs-Kontroll-Modell, meist mit dem englischen Begriff „Job Strain“ (dt. etwa berufliche Belastung) bezeichnet. Job Strain erzeugt physiologisch und mental chronischen Stress. Soziale Unterstützung bei der Arbeit kann die Effekte von Arbeitsstress abpuffern [10].
- Ein Missverhältnis von beruflicher Anstrengung und beruflicher Belohnung in der Wahrnehmung der betroffenen Person zeichnet das Modell beruflicher Gratifikationskrisen („effort-reward-imbalance“) aus. Belohnungen in dem Sinne sind z. B. Anerkennung, Verdienst, Lern- und Aufstiegschancen oder Arbeitsplatzsicherheit. Die individuelle Neigung zu Überengagement („overcommitment“) erhöht das Risiko für Gratifikationskrisen, weil die Erwartung an die Belohnung dieser Leistungen entsprechend hoch ist. Die gesundheitsschädliche Wirkung von Gratifikationskrisen wird durch die Verletzung der grundlegenden Norm sozialer Reziprozität erklärt [11].
- Verteilungsgerechtigkeit, prozedurale und Beziehungsgerechtigkeit konstituieren das Modell der Organisationsgerechtigkeit. Mit prozeduraler Gerechtigkeit wird die Entwicklung und die Einhaltung vereinbarter innerbetrieblicher fairer Regeln unter Einbezug der Beschäftigten verstanden, unter Beziehungsgerechtigkeit Fairness und Respekt, v. a. vonseiten der Führungskräfte [12].
- Unsicherheit hinsichtlich Veränderungen oder Verlust des Arbeitsplatzes („job insecurity“) stellt auf mehrfache Weise ein Gesundheitsrisiko für Betroffene dar, indem die drohende Änderung u. U. die soziale Identität und die ökonomische Lage der betroffenen Person bedroht [13, 14].

Basierend auf einer enormen Fülle von Studien ist es gesichertes Wissen, dass diese und andere spezifische, klar definierte und über Fragebögen messbare Konstellationen zu psychischen und psychosomatischen Erkrankungen beitragen, v. a. bei Depressionen und kardiovaskulären Erkrankungen. Beispielhaft seien hier aktuelle Metaanalysen (bzw. Meta-Metaanalysen; [14, 15]) angeführt, die insbesondere hohe Belastung am Arbeitsplatz und lange Arbeitszeiten als Risikofaktor für koronare Herzkrankheiten, (ischämischen) Schlaganfall und Depression identifizieren. Seidler und Kolleg:innen [16] errechneten sogar eine Verdopplung des Risikos für eine depressive Störung unter hohem Job Strain, also hoher Anforderung bei niedriger Kontrolle. Aus dem relativen Risiko, durch Arbeitsstress zu erkranken, und der Prävalenz von Arbeitsstress in der berufstätigen Bevölkerung lässt sich errechnen, welche Anteile des Krankheitsgeschehens auf Arbeitsstress zurückzuführen sind. In Europa sind dies insgesamt, d. h. unter Berücksichtigung aller Formen von Arbeitsstress, zwischen 17 % und 35 % der Depressionen und zwischen 5 % und 11 % der koronaren Herzerkrankungen, die durch berufliche Einflüsse bedingt sind [15]. Das muss für die Behandlung dieser Erkrankungen Konsequenzen haben.

Auf *Ebene des Betriebs* kann durch Verminderung von Arbeitsstress im o. g. Sinne und die gesundheitsförderliche Gestaltung von Arbeitsbedingungen das Erkrankungsrisiko reduziert werden, wie ebenfalls zahlreiche Interventionsstudien belegen [17, 18]. Hier scheinen v. a. Beteiligung und Einflussmöglichkeiten der Beschäftigten hinsichtlich der Gestaltung ein wesentlicher Erfolgsfaktor zu sein [17]. Entsprechend sind Arbeitgeber nach dem Arbeitsschutzgesetz verpflichtet, Gefährdungen durch psychische Belastungen der Arbeit zu erfassen, Gegenmaßnahmen zu planen, diese umzusetzen und die Wirksamkeit zu überprüfen [19]. Dieser Verpflichtung kommen aber nur wenige Arbeitgeber überhaupt und noch weniger in zureichendem Maße nach [20].

Auf *Ebene der individuellen Person* sind die Ansatzpunkte frühes Erkennen, rasche Behandlung und schnelle Wiedereingliederung von Erkrankenden bzw. Erkrankten im Betrieb (Sekundär- und Tertiärprävention). Das individuelle Vorgehen steht in Wechselwirkung zur Gefährdungsminimierung bzw. Arbeitsgestaltung: Einerseits werden belastende Arbeitsbedingungen in der Regel bei besonders vulnerablen Individuen am ehesten zu Gesundheitsstörungen führen; diese individuelle Komponente hat in der betrieblichen Gefährdungsbeurteilung, die sich laut Gesetz primär auf die Arbeitsbedingungen, nicht auf die Beanspruchung von einzelnen Beschäftigten konzentriert, aber wenig Raum. Andererseits führen auch Erkrankungen, die unabhängig von Arbeitsbedingungen entstehen, u. U. zu erheblichen Schwierigkeiten am Arbeitsplatz, was einen Teufelskreis erzeugen kann. Schließlich, und das kann entscheidend sein, ist der Erfolg von sekundär- und tertiärpräventiven Maßnahmen davon abhängig, inwieweit Arbeitsstress, der ggf. ursächlich zur Erkrankung beigetragen hat, verändert werden kann.

Präventives Eingreifen in diese Wechselwirkungen an der Schnittstelle zwischen Arbeitswelt und gesundheitlichem Versorgungssystem erfordert das Zusammenwirken vieler Akteure: Arbeitgeber, die Personen, an die Verantwortung für die Gefährdungsbeurteilung psychischer Belastung delegiert worden ist (typischerweise Führungskräfte), Fachkräfte für Arbeitssicherheit und Betriebsärzt:innen, Sozialdienste oder entsprechende „Kümmerer“ im Betrieb auf der einen, Fachleute mit psychiatrischer, psychosomatischer und psychotherapeutischer Expertise andererseits. Betriebliche Akteure können z. B. ihre Kenntnisse über die betrieblichen, äußeren Belastungsfaktoren wie den o. g. Arbeitsstress, aber auch über Ressourcen wie soziale Unterstützung am Arbeitsplatz in das Geschehen einbringen. Die therapeutischen Fächer konzentrieren sich mehr auf die individuelle, innere Sichtweise der betroffenen Person. Hier ergibt sich eine Erfolg versprechende Synergie, wenn beides, äußere Bedingungen und inneres Erleben, in den Blick genommen und Ziel von Veränderungen wird.

## Implementierung der Psychotherapeutischen Sprechstunde am Arbeitsplatz

### PT-A am Universitätsklinikum Ulm

Aus dem eben Gesagten geht hervor, dass für den Erhalt bzw. die Wiederherstellung der Arbeitsfähigkeit ein rasches, niedrigschwelliges Hilfsangebot sinnvoll ist und während der Behandlung auch der Arbeitsplatz als wichtiger Faktor bei der Entstehung und Aufrechterhaltung von psychischen Störungen einbezogen werden sollte. Die Klinik für Psychosomatische Medizin und Psychotherapie am Universitätsklinikum Ulm hat beispielsweise ca. 2011/2012 mit der Einrichtung einer PT‑A begonnen und betreut aktuell 23 Betriebe und öffentliche Einrichtungen. Die konkreten Versorgungsmodelle sind dabei jeweils individuell mit den jeweiligen Betrieben vereinbart. Zum Teil finden die Erstgespräche in den Räumen der jeweiligen Einrichtung statt, überwiegend in den Räumen unserer Klinik. Zum Teil können sich Betroffene, anonym für den Arbeitgeber, melden, z. T. erfolgt eine Vermittlung in unsere Sprechstunde über den zuständigen Betriebsarzt, in Einzelfällen auch über den betrieblichen Sozialdienst. Der Umfang des im Rahmen der Sprechstunde vom Auftraggeber finanzierten Stundenkontingentes variiert dabei zwischen 3 (mehr oder weniger vertiefte Diagnostik) bis 13 Sitzungen (schon Kurzzeittherapie). Knapp die Hälfte aller Betriebe ermöglicht 10–12 Sitzungen. Das durchschnittliche Kontingent liegt bei 7,6 Sitzungen. Im Schnitt aller Kontakte nehmen die Klienten 3,5 Sitzungen wahr, häufiger in größeren zeitlichen Abständen.

### Integrierte Versorgung – ein spezifisches Modell der PT-A

Neben dem sehr flexiblen Modell der PT‑A wird im Folgenden ein spezifisches, von einzelnen Krankenkassen finanziertes Modell vorgestellt: In verschiedenen Regionen (z. B. in Nord-Bayern und Ost-Niedersachsen) wurden Verträge zur Integrierten Versorgung (§ 140a bis d SGB V) abgeschlossen. Damit soll die interdisziplinäre Zusammenarbeit zwischen Betriebskrankenkassen (BKKen) und Arbeitsmediziner:/Psychiater: und Psychotherapeut:innen gefördert und eine diagnostische Abklärung sowie indizierte Behandlung von Arbeitnehmer:innen (und ggf. ihren Angehörigen) zeitnah ermöglicht werden. Gleichzeitig werden in der ambulanten Regelversorgung bestehende Hürden (u. a. lange Wartezeiten, ungenügende Validität der Diagnosen und Fehlbehandlungen) adressiert [21].

#### Ablauf.

Bei Verdacht auf eine psychische Störung, z. B. aufgrund hoher Fehlzeiten oder Schwierigkeiten am Arbeitsplatz, können Arbeitsmediziner:innen der:dem Arbeitnehmer:in die Teilnahme an der Integrierten Versorgung vorschlagen. Mitarbeiter:innen können sich auch direkt bei der:dem Fallmanager:in der zuständigen BKK anmelden. Die Teilnahme ist in jedem Fall freiwillig. Zunächst erhalten Teilnehmende eine sogenannte diagnostische Beratung. Stellt sich in diesem Rahmen heraus, dass eine psychopharmakologische und/oder psychotherapeutische Behandlung indiziert ist, wird diese zeitnah und ohne Gutachterverfahren in Psychotherapieambulanzen oder bei vertraglich gebundenen Psychotherapeut:innen initiiert. Zunächst wird eine Kurzzeittherapie mit einem Umfang von 25 Sitzungen überwiegend in Form einer kognitiven Verhaltenstherapie veranschlagt. Ist eine Verlängerung nötig, kann diese durch einen Bericht über den bisherigen Verlauf und Begründung der Fortsetzungsnotwendigkeit in eine Langzeittherapie umgewandelt werden. Falls vor einer ambulanten psychotherapeutischen Behandlung eine stationäre Behandlung nötig erscheint, z. B. bei zusätzlichen psychischen Störungen oder internistisch zu behandelnden Erkrankungen, bestehen weitere Verträge zwischen den Betriebskrankenkassen (BKKen) und anderen Einrichtungen (u. a. einer Klinik für Psychiatrie und Psychotherapie bzw. Psychosomatische Rehabilitation). Nach Abschluss einer stationären Behandlung soll sichergestellt werden, dass die Patient:innen innerhalb weniger Werktage die ambulante Behandlung weiterführen können.

#### Rahmenbedingungen.

Wichtige betriebliche Akteure werden vor Beginn der Implementierung ausführlich schriftlich und persönlich informiert (z. B. Zuständige des Eingliederungsmanagements, Sozialdienst, Suchtbeauftragte); Bedenken müssen in jedem Fall ausgeräumt werden. Die Arbeitsmediziner:innen sind zentrale Ansprechpartner:innen im Betrieb für Arbeitnehmer:innen und Multiplikator:innen. Daher sollten diese in motivierender Gesprächsführung geschult sein und die psychosomatische Grundversorgung erbringen können. Um anschließend ein lückenloses und zeitsparendes Fallmanagement zu gewährleisten, muss ein engagierter und andauernder Austausch zwischen den Kooperationspartner:innen gepflegt werden. Idealerweise übernehmen geschulte Fallmanager:innen die Aufgabe, die Betroffenen umfassend zu betreuen und zu beraten sowie zeitnah Kontakt zur Ambulanz aufzunehmen. Dort gewährleisten Fachkräfte zur Qualitätssicherung die Übermittlung von Berichten und Befunden, damit ggf. indizierte Maßnahmen schnell in die Wege geleitet werden können und keine zeitlichen Verzögerungen entstehen. Der bürokratische Aufwand für die Betroffenen und Behandler:innen wird hierdurch minimiert.

#### Diagnostische Beratung.

Spezifisch ausgearbeitete Schweigepflichtsentbindungen sind die Voraussetzung für die Kontaktaufnahme und den Informationsaustausch mit den Arbeitsmediziner:innen, den Fallmanager:innen und den heilkundlich Tätigen. Arbeitnehmer:innen, die sich für eine Teilnahme an der Integrierten Versorgung entschieden haben, nehmen zunächst an der diagnostischen Beratung in der zuständigen Psychotherapieambulanz teil. Diese umfasst in der Regel 3 Sitzungen, in denen ein ausführliches Erstgespräch, eine Anamnese der aktuellen Beschwerden und der Situation am Arbeitsplatz sowie eine ausführliche Erhebung des psychischen Befunds erfolgen. Zudem werden standardisierte Selbstbeurteilungsinstrumente in computerisierter Form durchgeführt. In der dritten Sitzung werden der:dem Patient:in die Ergebnisse ausführlich dargelegt, es werden eine psychoedukative Beratung durchgeführt und ggf. weitere Maßnahmen (z. B. Physiotherapie, Zielgespräch mit dem Vorgesetzten, Schuldnerberatung) empfohlen. Dabei werden bereits während der diagnostischen Beratung erste Schritte eingeleitet (z. B. Erarbeitung von Regeln zur Schlafhygiene, Aufbau angenehmer Aktivitäten, Anleitung von Entspannungsverfahren). Mit dem Einverständnis der Patient:innen werden die erarbeiteten Empfehlungen schriftlich an andere Behandler:innen übermittelt. Liegt eine psychische Störung vor, so gilt die Stellungnahme des:der beratenden Therapeut:in für die Zuständigen der BKKen als Therapieempfehlung. Sofern die:der Arbeitnehmer:in eine Behandlung wünscht, wird innerhalb von 15 Werktagen ein Therapiebeginn ermöglicht. Damit werden die Voraussetzungen für arbeitsplatzbezogene Ansätze der Psychotherapie gelegt [22].

#### Psychotherapeutische Behandlung.

Bei den häufig vorkommenden Angst- und affektiven Störungen bietet die Psychotherapie nicht nur eine effektive, sondern auch effiziente Behandlungsmöglichkeit [23]. In einer Datenanalyse von BKKen zeigte sich zudem, dass kognitiv-behaviorale und tiefenpsychologisch fundierte Therapien zu einer Reduktion von Arbeitsunfähigkeitstagen beitragen können [24]. Werden Interventionen integriert, die den Arbeitsplatz fokussieren, dürfte die Arbeitsfähigkeit eher wiederhergestellt werden: So konnte eine arbeitsplatzbezogene kognitive Verhaltenstherapie die Rate der Arbeitsunfähigkeitstage bei Erwerbstätigen mit depressiver Episode stärker als die herkömmliche Verhaltenstherapie reduzieren [25].

Neben einem störungsspezifischen Vorgehen sollte der Arbeitsplatz frühzeitig thematisiert und – wenn notwendig – die betriebliche Eingliederung vorbereitet werden [26]. So können beispielsweise übliche Störungsmodelle durch arbeitsbezogene Modelle ergänzt, Indikationen für spezifische Maßnahmen erarbeitet und Pläne für eine schrittweise Arbeitswiederaufnahme gemeinsam mit der:dem Patient:in erstellt werden. Strategien zur erfolgreichen Bewältigung des Arbeitsbeginns können erarbeitet oder mögliche Hindernisse (z. B. mithilfe von Rollenspielen) ausgeräumt werden. Falls notwendig, werden Kontakte zum betrieblichen Eingliederungsmanagement hergestellt. In Absprache mit den Arbeitsmediziner:innen können Interventionen auch direkt am Arbeitsplatz durchgeführt werden, z. B. zur Optimierung der Arbeitsorganisation.

## Betriebsnahe Versorgungsnetzwerke im Sinne einer PT-A – wissenschaftlicher Kenntnisstand

Die hier vorgestellten Modelle einer PT‑A, welche Synergien verschiedener ärztlicher und psychotherapeutischer Professionen aufnehmen bzw. sektorenübergreifend oder interdisziplinär vorgehen, erscheinen als ein wirkungsvolles Instrument zur Gesunderhaltung und Stabilisierung der Arbeitsfähigkeit Erwerbstätiger. Vereinzelt ist es bereits gelungen, Kooperationen dieser Art sozialrechtlich abzubilden, wie im vorangehenden Abschnitt (mit Modellen der Integrierten Versorgung) darlegt wurde.

Modelle dieser Art sind notwendig, da Interventionen, die rein die individuelle Symptomebene adressieren, die klinische Einschränkung zwar reduzieren, die Teilhabefähigkeit der Behandelten jedoch nicht verbessern [27]. Interventionen, die an den Arbeitsverhältnissen ansetzen, sind primärpräventiv wirksam, in Bezug auf bereits Erkrankte zeigen sie jedoch keinen Effekt [28, 29]. Integrierte Modelle hingegen belegen einen Effekt sowohl auf der Symptomebene als auch in Bezug auf die Teilhabefähigkeit [30, 31]. Diese Modelle sind heterogen, was den national differierenden Sozialversicherungssystemen und -Gesetzgebungen geschuldet ist sowie dem Pilotcharakter solcher Initiativen, die vor allem durch die lokalen Bedingungen und Anforderungen geformt sind.

In dieser Tradition entstanden in Deutschland, wie eingangs berichtet, u. a. zunächst die „Psychosomatische Sprechstunde im Betrieb (PSIB)“ [32] im süddeutschen Raum, wie auch die oben beschriebene „Diagnostische Beratung (DB)“ [21] im Norden. Die Weiterentwicklung aus den wirksamen Komponenten einiger dieser Modelle unter Einbezug aktueller Forschung wurde umbenannt in PT‑A. Die PT‑A basiert auf Daten zu Nutzergruppen [33, 34] und Anlässen der PSIB [35], qualitativ ermittelten Wirkfaktoren und Spannungsfeldern durch das neue Modell [36–38], ersten Belegen für die Wirksamkeit unter Routinebedingungen [34, 39–41], positiven Ergebnissen zur Nutzerzufriedenheit [34], der Expertise und Ergebnissen aus der DB [21] sowie aus Daten zu betriebsnahen Versorgungsnetzwerken [42, 43], den bereits als wirksam erwiesenen arbeitsbezogenen Interventionen [25] und der PSIB [32, 44]. Die PT‑A ist ein ergänzendes Angebot zur psychotherapeutischen Regelversorgung, das eine niedrigschwellige und kurzfristige psychotherapeutische Unterstützung für Beschäftigte mit subklinischen oder ausgeprägten psychischen Beschwerden bereitstellt [45].

## Die Studie „Frühe Intervention am Arbeitsplatz“ (friaa)

Ziel der PT‑A ist es, Symptome zu reduzieren, die Arbeitsfähigkeit zu sichern, eine schnellere Rückkehr zur Arbeit zu erreichen bzw. das Risiko für eine Erwerbsminderung zu senken. Diese Annahmen werden aktuell im Rahmen der Studie „Frühe Intervention am Arbeitsplatz“ (friaa) mittels einer multizentrischen randomisiert kontrollierten Studie (RCT) zur Implementierung und Untersuchung der Wirksamkeit einer PT‑A in Deutschland untersucht (Laufzeit 06/2020 bis 09/2024; [46]). Im Rahmen der Studie erhielten psychisch beanspruchte Beschäftigte ein Erstgespräch mit ausführlicher klinisch-psychologischer Diagnostik und eine entsprechende Befundrückmeldung mit (Weiter‑)Behandlungsempfehlung. Teilnehmende der Interventionsgruppe erhielten darüber hinaus bis zu 16 psychotherapeutische Folgegespräche [46].

### Studiendesign und Studienpopulation.

Zwischen September 2021 und Januar 2023 konnten Arbeitnehmer:innen aus 54 kooperierenden Betrieben aus verschiedenen Regionen Deutschlands mit insgesamt über 125.000 Beschäftigten am RCT teilnehmen. Außerdem gab es Kooperationen mit 11 überbetrieblichen Multiplikator:innen, sodass auch Beschäftigte von Betrieben die Möglichkeit hatten, die durch diese Multiplikator:innen betreut wurden. Einschlusskriterium für die Betriebe war, dass sie während der Laufzeit der Intervention den eigenen Beschäftigten kein vergleichbares Angebot zur psychotherapeutischen Unterstützung anboten. Im Zeitraum von Juli 2022 bis Dezember 2022 wurden zusätzlich rund 55.000 Personen in den Regionen Düsseldorf, Ulm und Hildesheim über die sozialen Plattformen Facebook und Instagram mit Werbeanzeigen auf die Studie aufmerksam gemacht. Weiterhin wurden in den Regionen Erlangen und Hildesheim jeweils 2 Artikel in lokalen Zeitungen veröffentlicht, um Beschäftigte anzusprechen. Teilnehmen konnten Beschäftigte zwischen 18 und 65 Jahren mit einer Arbeitszeit von mind. 15 Wochenstunden, die klinische Symptome einer psychischen Erkrankung der sogenannten „common mental disorders (CMD)“ kodiert nach ICD-10 (F32–34, F42, F43, F45, F48, F51) aufwiesen oder Symptome einer psychosomatischen Erkrankung ohne ICD-10-Diagnose gemessen mit dem Global-Assessment-of-Functioning-Fragebogen (GAF) mit einem Wert von < 81 [47, 48]. Weitere Informationen zum Studiendesign und zur Studienpopulation können dem Studienprotokoll entnommen werden [46]. Darüber hinaus wurden die betrieblichen Ansprechpersonen der 54 kooperierenden Betriebe (z. B. Betriebsarzt/Betriebsärztin, BEM-Mitglied) und 11 Multiplikator:innen, die die Implementierung und die Vermittlung in die PT‑A unterstützten, zur Beantwortung eines Online-Fragebogens zur Implementierung der PT‑A eingeladen.

Im vorliegenden Themenheft werden bereits erste Daten der Baseline-Erhebung (von 550 eingeschlossenen Beschäftigten vor Randomisierung in Kontroll- oder Interventionsgruppe) publiziert. Ergebnisse werden unter anderem im Artikel von *Kohl et al.* berichtet. Erste Daten zu gesundheitsökonomischen Aspekten berichten *Mulfinger et al.*, wohingegen *Hander et al.* das Nutzer:innenprofil in Bezug auf Unterschiede bezüglich der Betriebsgröße diskutieren. *Herold und Kolleg:innen* beleuchten Aspekte zur Migrationsgeschichte in Bezug auf besondere Herausforderungen in der Arbeitswelt.

## Fazit

Die PT‑A ist eine vielversprechende Einrichtung, um die Last durch psychische Störungen und psychosomatische Erkrankungen bei Erwerbstätigen zu verringern. Ob sich diese Erwartung erfüllt, werden die Ergebnisse der laufenden randomisierten kontrollierten Studie *friaa* zeigen.

## Footnotes

1 „Psych*-Fächer“: Psychiatrie, Psychosomatik, Kinder- und Jugendpsychiatrie.
